# Intestinal Intraepithelial Lymphocyte-Enterocyte Crosstalk Regulates Production of Bactericidal Angiogenin 4 by Paneth Cells upon Microbial Challenge

**DOI:** 10.1371/journal.pone.0084553

**Published:** 2013-12-17

**Authors:** Catherine R. Walker, Isabelle Hautefort, Jane E. Dalton, Karin Overweg, Charlotte E. Egan, Roy J. Bongaerts, Darren J. Newton, Sheena M. Cruickshank, Elizabeth M. Andrew, Simon R. Carding

**Affiliations:** 1 Gut Health and Food Safety, Institute of Food Research, Norwich, United Kingdom; 2 Faculty of Biological Sciences, University of Leeds, Leeds, United Kingdom; 3 Centre for Immunology and Infection, University of York, York, United Kingdom; 4 Pediatric Surgery, Children’s Hospital of Pittsburgh, UPMC, Pittsburgh, Pennsylvania, United States of America; 5 Leeds Institute of Cancer & Pathology, Wellcome Trust Brenner Building, St James’s University Hospital, Leeds, United Kingdom; 6 Faculty of Life Sciences, University of Manchester, Manchester, United Kingdom; 7 Norwich Medical School, University of East Anglia, Norwich, United Kingdom; Massachusetts General Hospital, United States of America

## Abstract

Antimicrobial proteins influence intestinal microbial ecology and limit proliferation of pathogens, yet the regulation of their expression has only been partially elucidated. Here, we have identified a putative pathway involving epithelial cells and intestinal intraepithelial lymphocytes (iIELs) that leads to antimicrobial protein (AMP) production by Paneth cells. Mice lacking γδ iIELs (TCRδ^-/-^) express significantly reduced levels of the AMP angiogenin 4 (Ang4). These mice were also unable to up-regulate Ang4 production following oral challenge by *Salmonella*, leading to higher levels of mucosal invasion compared to their wild type counterparts during the first 2 hours post-challenge. The transfer of γδ iIELs from wild type (WT) mice to TCRδ^-/-^ mice restored Ang4 production and *Salmonella* invasion levels were reduced to those obtained in WT mice. The ability to restore Ang4 production in TCRδ^-/-^ mice was shown to be restricted to γδ iIELs expressing Vγ7-encoded TCRs. Using a novel intestinal crypt co-culture system we identified a putative pathway of Ang4 production initiated by exposure to *Salmonella*, intestinal commensals or microbial antigens that induced intestinal epithelial cells to produce cytokines including IL‑23 in a TLR-mediated manner. Exposure of TCR-Vγ7^+^ γδ iIELs to IL-23 promoted IL‑22 production, which triggered Paneth cells to secrete Ang4. These findings identify a novel role for γδ iIELs in mucosal defence through sensing immediate epithelial cell cytokine responses and influencing AMP production. This in turn can contribute to the maintenance of intestinal microbial homeostasis and epithelial barrier function, and limit pathogen invasion.

## Introduction

The gastrointestinal (GI) tract is a focal point of microbial/host interactions, accommodating the vast resident microbiota, yet protecting against invasion by microbial pathogens. Protection of the intestinal mucosa is multilayered comprising physical, chemical and immunological mediators that collectively contribute to the development of innate immune responses and maintenance of intestinal homeostasis [1]. Prominent among chemical defences in the small intestine are antimicrobial proteins (AMPs) such as defensins (cryptdins in mice), lysozyme and Angiogenin 4 (Ang4) that are produced by Paneth cells residing at the base of the crypts. AMPs have a broad activity against Gram‑positive and ‑negative bacteria, protozoa and fungi [2,3]. AMP-deficient mice are more susceptible to bacterial infection [4], and over-expression of human defensins in transgenic mice increases resistance to pathogen invasion [3]. AMPs are therefore assumed to play a central role in preventing pathogen invasion and constraining proliferation of commensal microorganisms in close vicinity to the epithelium [5]. Since only few direct molecular pathways of microbe-induced AMP production have been defined, the possibility exists that other locally produced factors influence Paneth cell AMP production. Potential sources of these factors and cytokines are the populations of mucosa-associated immune cells including intestinal intraepithelial lymphocytes (iIELs) [6], B‑1 B cells [7], mucosa-associated invariant T (MAIT) cells [8], and various innate lymphoid cells (e.g. NK cells, lymphoid tissue inducer cells and nuocytes) distinguished on the basis of their functional characteristics and cytokine profile [9,10]. Of these immune cell populations, iIELs are the closest populations of immune cells to the host-lumen interface, residing between the basolateral faces of adjacent epithelial cells, making them ideally suited to interact with epithelial cells and influence their response to microbial challenge [11,12].

iIELs are found in the majority of mammals and birds [12-16] and are enriched in lymphocytes expressing the γδ T cell receptor (TCR). In most inbred strains of mice T cells expressing Vγ7 or Vγ1 encoded TCRs make up to 90% of γδ iIELs, among which Vγ7^+^ cells comprises the largest population [17,18]. The biological function of iIELs is still unclear although studies carried out primarily in mice demonstrating their inherent cytotoxicity [19], their capacity to produce pro-inflammatory cytokines, epithelial cell growth factors and AMPs [20], their involvement in epithelial restitution [21] and maintenance of epithelial tight junction complexes [22] suggest they play a central role in intestinal defence [21,23-25]. 

Here we have investigated the role that γδ iIELs play in the immediate response to oral infectious challenge and in particular, in influencing Paneth cell production of AMPs. 

### Study rationale

γδ iIELs dominate iIEL populations in mammals [12,13,15] and their absence or depletion in mice is associated with increased susceptibility to infection by enteric pathogens [26,27]. Here we tested the hypothesis that γδ iIELs contribute to gut barrier functions by rapidly influencing local AMP production in response to microbial challenge using *Salmonella enterica* serovar Typhimurium as a model bacterium to trigger host innate intestinal antimicrobial responses. The approach was to capture events occurring *in vivo* immediately and within 2 hours of oral microbial challenge as iIELs are fast‑acting cells [28,29], and Paneth cells release pre-formed antimicrobial proteins from their granules within minutes of exposure to appropriate inflammatory stimuli [2].

## Materials and Methods

### Mice and infections

Six to ten week old C57BL/6J (Harlan Labs), C57BL/6J-TCRδ^-/-^ (JAX Laboratories) and C57BL/6J-TCRVγ1^-/-^ [30] were housed in a conventional animal facility at the Universities of Leeds and East Anglia. Mice were challenged using isolated intestinal loops [31] or by oral gavage with 4x10^8^ of viable or killed invasive WT SL1344 [32] and non-invasive (ΔSPI-1) *Salmonella enterica* serovar Typhimurium (*S*. Typhimurium) SL1344 [33] or 4x10^8^ invasive *Salmonella* expressing a luciferase (*lux*) reporter gene (SL1344-Tn5*lux*) [34]. Bacterial CFU in the intestinal mucosa was determined after PBS rinse of intestinal tissue by plating serial dilutions of tissue homogenates on BHI, and LB (Oxoid) agar plates. In the case of SL1344‑Tn5*lux*, luminescent bacterial colonies were also counted (IVIS 100, Xenogen Corp.). 

### Ethics Statement

Protocols for all the animal experiments included in this manuscript were conducted in full accordance with the Animal Scientific Procedures Act 1986 under Home Office approval. Mice were euthanized by Schedule One approved methods, and all efforts were made to minimize animal suffering.

### iIELs culture

iIELs were isolated from Peyer’s patch-excised small intestines [35]. Highly purified preparations of Vγ7^+^ and Vγ7^-^ iIELs were obtained by staining iIELs from WT mice with F(ab’)_2_ fragments of anti-TCRδ (clone GL3), CD3 (H57-597) and ‑TCR-Vγ7 (F2.67) antibodies and sorting on an inFlux High Speed Cell Sorter (Becton Dickinson); isolated cells were routinely >98% TCRγδ^+^ and >95% viable. iIEL-conditioned medium was obtained by incubating 3x10^4^ iIELs with phorbol myristate acetate (PMA) and ionomycin (Io) or anti-CD3 (UCHT-1, Becton Dickinson) and anti-CD28 (CD28.2, Becton Dickinson) antibodies at 37°C in 5% CO_2_ for 16h. For adoptive transfer, 5-8 x10^5^ FACS‑isolated iIELs were injected i.v. into C57BL/6-TCRδ^-/-^ mice. Five to six weeks later these mice were orally challenged with *S*. Typhimurium (SL1344) as described above. Two hours later intestines were removed for immunohistochemical analysis and/or bacterial counts. γδ iIEL reconstitution was verified by staining sections of intestine with anti-CD3, -TCRγδ and ‑TCRVγ7 antibodies and counting the number of stained cells per villus in at least 30 *villi* per section on a minimum of 5 sections per tissue sample from ≥4 mice.

### Intestinal crypt and epithelial cell culture

Crypts were isolated from fragments of small intestine by sequential incubation with 30mM EDTA, 10% FCS (Biosera) and 1mM DTT (adapted from [2,36]) and identified by their morphology, phloxine-tartrazine staining, expression of Ki-67 and lack of alkaline phosphatase activity. 500 to 2x10^4^ crypts were cultured in iPIPES (10mM PIPES pH7.4 containing 137mM NaCl) with or without secretory stimuli. Stimuli (10μM CCh, 10^3^ *Salmonella*, 100ng/ml IL-22) were added for 30 min at 37°C. Crypt exudates were obtained by incubating crypts in iPIPES at 37°C, 5% CO_2_ for 3h followed by centrifugation for 5 min at 150g, and used in killing assays. The average volume of a small intestinal crypt of 120μm in height was calculated to be 13pl using the method of Ayabe [2]. Monolayer cultures of intestinal epithelial cells were obtained as described previously [37]. For co-culture with iIELs in iIEL-conditioned medium and recombinant cytokines, crypts were cultured at 37°C for 4h in complete tissue culture media. In some experiments, anti-IL‑22 neutralising antibodies (1.5μg/ml) or isotype control antibodies (1.5μg/ml) were added. Supernatants were collected and stored at -20°C and cells were harvested for mRNA extraction. Culture supernatants were analysed for lysozyme activity using the EnzChek lysozyme assay kit (Invitrogen).

Polarised monolayers of intestinal epithelial cells were challenged with 1-5x10^6^ CFU wild type or specific mutants of the S. Typhimurium strain SL1344 [31] for up to 16h. For challenge with microbial antigens, cells were incubated with 10μg/ml peptidoglycan (*S. aureus*), 1μg/ml lipopolysaccharide (*S*. Typhimurium), 1μg/ml muramyl dipeptide or 500ng/ml CpG for up to 16h in 5% CO_2_ at 37°C. Supernatants were analysed for protein production by ELISA and cell pellets for cytokine/Ang4 mRNA expression by qPCR. Lamina propria cells were isolated on ice from Peyer’s patch‑excised and longitudinally cut small intestines of WT mice. The epithelial layer was removed by incubation at 37°C for 20 min in RPMI containing 10% FBS, 5mM EDTA and 1mM DTT. Remaining tissue was washed in RPMI and then digested at 37°C for 1h under agitation (200rpm/min) in RPMI containing 10% FBS, 2mg/ml Collagenase D and 1mg/ml Dispase. Lamina propria cells were further enriched by Percoll gradient (40-70%) centrifugation with cell purity confirmed by staining with anti‑CD45 antibodies (FITC/PE‑conjugated (Ly-5), e‑Biosciences Ltd). *In vitro* Salmonella infection was performed using a multiplicity of infection of 10:1 (10 bacterial cell per lamina propria cell). At the end of the experiment, cells were harvested and RNA was purified with Tri‑reagent, reverse transcribed and analysed for IL‑23 mRNA expression by qPCR. 

### Cell line culture

*In vitro* control experiments were carried out on the mouse trans‑immortalised cell line of intestine epithelial cells, m‑ICc12 [38] and on the mouse tumour‑derived macrophages, RAW264.7 (ATCC® TIB71™), cultivated to a density of 1 and 6-8x10^6^ cells, respectively , in T25 flasks and infected with *S*. Typhimurium SL1344 at a multiplicity of infection of 10:1 and 100:1, respectively [21,39,40] or with gut commensal isolates grown overnight and used at a multiplicity of infection of 10:1. The murine commensals tested were *Escherichia coli*, *Enterococcus gallinarum*, both isolated from the caecum of C57BL/6J mice (IFR GH collection strains GH260 and GH76 GenBank JN412816, respectively), and human gut isolates *Bifidobacterium longum* (DSM20219) and *Bacteroides thetaiotaomicron* VPI‑5482 (DMSZ collection). Epithelial cells were harvested and RNA purified and processed as mentioned above. For *in vitro* TLR‑mediated challenge with *Salmonella*, 1x10^6^ epithelial cells were incubated with 1‑5x10^6^ CFU wild type or specific mutants of the *Salmonella* strain SL1344 [31] for up to 16 h. For challenge with microbial antigens intestinal epithelial cells were incubated with 10μg/ml peptidoglycan (*S. aureus*), 1μg/ml lipopolysaccharide (*S*. Typhimurium), 1μg/ml muramyl dipeptide or 500ng/ml CpG for up to 16 h in 5% CO_2_ at 37°C. Supernatants were analysed for protein production by ELISA, and cell pellets for cytokine/Ang4 mRNA expression by qPCR.

### Transmission electron microscopy

*Salmonella* cells were harvested, after treatment in 3% glutaraldehyde (Agar Scientific, UK), in 0.1M cacodylate buffer (pH 7.2) for 3h, washed three times in 0.1M cacodylate buffer (pH 7.2) and centrifuged. The cell pellets were embedded in molten 2% low-melting-point agarose (TypeVII, Sigma) that were sectioned, fixed in 2% aqueous osmium tetroxide for 2h then dehydrated three times through an ethanol series (10-100%). Samples were immersed in 1:2 mix of LR White medium grade resin (London Resin Company Ltd) and 100% ethanol for 18h followed by sequential 6h impregnation in 1:1 and a 2:1 mix of LR White resin and 100% ethanol. Samples were then bathed three times for 6h in 100% resin. Resin blocks from each sample were put into individual gelatine capsules with fresh resin and polymerised for 18h at 60°C. Ninety nm thick sections were cut using an ultramicrotome (Ultracut E, Reichert-Jung) with a glass knife, collected on film/carbon coated copper grids, and stained sequentially with uranyl acetate (saturated in 50% ethanol) and Reynold’s lead citrate. Sections were examined and imaged in a FEI Tecnai G2 20 Twin transmission electron microscope at 200kV.

### Microarray

For microarray analysis RNA was isolated from small intestinal epithelium of wild type, TCRδ^-/-^ and TCRVγ1^-/-^ mice (n=4) at 2h post infection with *Salmonella* and processed using the GeneChip Mouse Genome 430A 2.0 array by the Univ. Manchester Microarray Core Facility according to standard protocols [41]. Gene expression values were normalised with gcRNA and anti-logged (average from 3 samples). Technical quality control was performed with dChip (V2005) (www.dchip.org; [42]) using the default settings. Background correction, quantile normalization, and gene expression analysis were performed using GCRMA in Bioconductor [43].

### Ang4

Recombinant Ang4 was produced as described previously [44] with purity assessed by SDS-PAGE, N-terminal sequencing and biological activity determined by RNase activity [45]. Ang4 immunoblotting of total cellular lysates of small intestinal mucosa was carried out as described previously [44]. Membranes were stripped and re-probed with an anti-GAPDH antibody. Ang4 microbicidal activity was tested against 1x10^5^‑1x10^6^ cells of *S*. Typhimurium (SL1334wt) grown to mid-exponential phase, washed in PBS and incubated with increasing concentrations of recombinant Ang4 (10, 28, 64 and 280μM), or in exudates of isolated crypt in iPIPES buffer [2] prepared as above for 60 min at 37°C. Viable and culturable counts were determined by plating serial dilutions of test suspensions on LB agar plates and counting CFU 16h later. 

### Flow Cytometry

iIELs were stained with different combinations of CD3 (clone 1452C11), TCRγ (GL3) and TCRαβ (H57-597) (Caltag Laboratories), IL-23R antibodies (Imgenex) and, anti-TCR-Vγ7 (F2.67) and TCR-Vγ1 (2.11) antibodies provided by L. Lefrançois (Univ. Connecticut, CT) and P. Pereira (Institut Pasteur, Paris, France), respectively. For intracellular cytokine staining surface stained cells were fixed, permeabilised, stained with PE-conjugated anti-IL-22, IL-17A or isotype matched control antibodies (R&D Systems) and analysed on a FACSCalibur flow cytometer (Becton Dickinson). For the assessment of the *in vitro* impact of Ang4 on *Salmonella* cell membrane integrity, bacterial cells were treated with Ang4 or control PBS as described above, stained with PI (1μg/ml) and analysed on a Cytomics FC500 MPL flow cytometer (Beckman Coulter). Data analysis was performed using Flow Jo v7.6.2 software (Tree Star, Inc.).

### ELISA

ELISAs were used to quantify IL-22 (R&D Systems) and IL-23 (Insight Biotechnology Ltd) in iIEL and epithelial cell conditioned medium. An Ang4 ELISA was developed using an antiserum (M20; Santa Cruz) for the capture antibody and a rabbit anti-Ang4 antiserum [44] for detection of bound Ang4. Recombinant Ang4 was used to establish a standard curve for determining concentrations in conditioned medium. Limit of detection was 100pg.

### Immunohistochemistry

Paneth cells were visualised in paraffin-embedded sections of intestine by staining with phloxine-tartrazine. For localisation of Ang4 protein in Paneth cells, frozen sections of small intestine were stained with rabbit anti-mouse angiogenin antiserum [44] in combination with anti-lysozyme antibodies (Dako Cytomation) conjugated in house to Alexa‑Fluor 488 (Molecular Probes). Biotinylated goat anti-rabbit antibody (Caltag-Medsystems) and Texas Red streptavidin conjugate (Calbiochem) were used as secondary reagents. Sections were counterstained with DAPI. All sections were mounted and viewed using an inverted Zeiss Axiovert 200M microscope using Axiovision image analysis software (Imaging Associates Ltd).

### ang4::luc reporter system

An 829bp fragment, containing sequences immediately upstream of the Ang4 transcription initiation start site was cloned into the luciferase reporter vector pGL3-basic (Promega, Southampton, UK) and used to transfect (Metafectene Pro; Biontex, Germany) confluent monolayers of m‑ICc12 cells. To control for differences in transfection efficiency, epithelial cells were co-transfected with a *Renilla* luciferase reporter gene containing plasmid (pRL-TK; Promega) in which expression is under the control of the Herpes simplex virus thymidine kinase (HSV-TK) promoter, which provides constitutive expression of the reporter gene in mammalian cells. Transfected cells were grown in 96-well plates with serum-free media in the absence or presence *Salmonella* or a range of concentrations of LPS or recombinant IL-22 for 4h after which luciferase activity was determined using the Dual-Glo Luciferase assay system (Promega) and Berthold Technologies Lumat LB 9507 luminometer. Results are expressed as the ratio of Ang4-reporter:*Renilla* luminescence, which was background-subtracted (non-transfected cells with Dual-Glo substrate) and where multiple plates were used, normalised to the ratio of control wells treated consistently on all plates.

### qPCR

RNA was extracted from highly purified preparations (>97% cytokeratin+, <2% CD45^+^) of intestinal epithelial cells [46] or (~40% CD45^+^) LP cells using Tri-reagent and reverse transcribed using ImpromII reverse transcriptase (Promega). cDNA was used for Taqman real time quantitative PCR using optimised primer sets (Applied Biosystems) for Ang4 (Mm01315577_s1), IL-23/p19 (Mm00518984_m1), IL-22 (Mm00444241_m1) and the reference gene, β-actin (Mm00607939_sl). Data was analysed using the ∆∆Ct comparative method. Results were expressed as relative expression to the β-actin reference gene or compared with control samples as stated.

### Statistical analysis

All data were assessed for normal distribution using a Shapiro-Wilk test. Kruskal Wallis with post Nemeyi-Damico-Wolfe-Dunns test and ANOVA with post-test of Bonferroni were also used when appropriate. For parametric data on experiments comparing two groups only, analysis was performed using a Student t‑test and for non-parametric data analysis was performed using Mann Whitney U tests using the Statistical Package for the Social Sciences software, SPSS (Surrey, UK). Statistical significance was taken as P<0.05.

## Results

### Mice lacking a subset of iIELs exhibit reduced levels of Ang4

**Figure 1:**
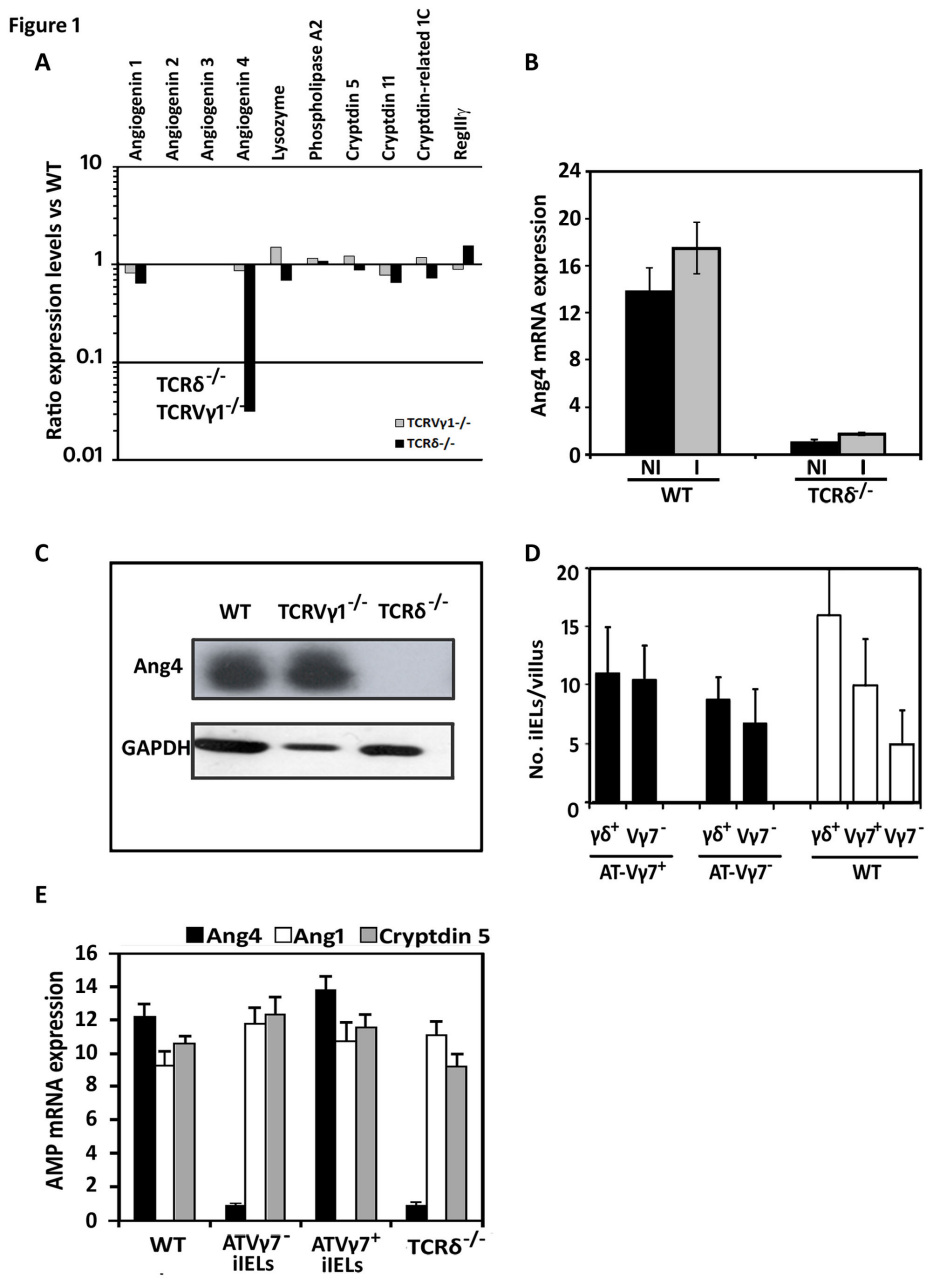
γδ T cells modulate the production of Ang4. (A) Expression levels of Paneth cell AMPs in the intestinal mucosa of naïve wild type (WT), TCRδ^-/-^ and TCRVγ1^‑/-^ mice as determined by RNA microarrays [82]. Levels are displayed as a ratio of the value obtained in TCRδ^-/-^ or TCRVγ1^-/-^ versus WT samples. (B) The level of Ang4 mRNA in the intestinal epithelium of TCRδ^-/-^ and WT mice was determined both prior to (NI) and 2h post-oral challenge and infection (I) with *Salmonella* Typhimurium by qPCR. Data (mean±SEM) are expressed relative to levels of β‑actin mRNA and were collated from 6 RNA samples in each group. (C) Ang4 protein levels in the intestine of naïve WT, TCRδ^‑/-^ and Vγ1^‑/-^ mice determined by immunoblotting. Membranes were stripped and re-probed with an anti-GAPDH antibody. The results shown are representative of those obtained using 4-6 mice of each strain. (D) Number of γδ^+^ and TCR-Vγ7^+^ iIELs in intestinal tissue sections by immunohistochemistry 6 weeks after transfusion of γδ iIEL-deficient mice with TCR-Vγ7^+^ or TCR-Vγ7^-^ iIELs. The data were collated (mean±SEM) by counting stained cells in at least 30 villi per section on a minimum of 5 sections per tissue from 4-6 mice. (E) Ang4 production is restored in iIEL-reconstituted TCRδ^‑/-^ mice to WT levels after transfusion of Vγ7^+^ (ATVγ7^+^) iIELs. Levels of Ang4, Ang1 and cryptidin 5 mRNA were determined by qPCR in samples of small intestine obtained from WT, TCRδ^-/-^ and TCRδ^-/-^ mice 6 weeks post‑reconstitution with Vγ7^+^ or Vγ7^-^ (ATVγ7^-^) iIELs and 2h after oral challenge with *Salmonella*. Data (mean±STD) are expressed relatively to levels of β‑actin mRNA and were collated from RNA samples of 4-6 mice of each group.

Global transcriptional changes in AMP expression associated with the absence of γδ iIELs were assessed by microarray-based mRNA profiling of the intestinal epithelium of naïve wild type (WT) mice, mice lacking all γδ T cells (TCRδ^-/-^) and mice lacking the TCR-Vγ1^+^‑subset of γδ T cells (TCRVγ1^-/-^) [30]. The profile of all but one AMP mRNA examined was comparable in all three strains of mice except for Ang4 that was expressed at significantly (p≤0.01) lower levels (up to ~30-fold) in naïve TCRδ^-/-^ mice (Figure 1A and B). Of note, the levels of RegIIIγ mRNA were similar in all three strains of mice despite γδ iIELs being a source of this lectin [20]; indicative perhaps of biological redundancy and the ability of other RegIIIγ-producing cells compensating for the absence of γδ T cells under the experimental conditions used here. Reduced baseline levels of Ang4 protein in intestinal tissue of naive TCRδ^-/-^ mice were confirmed by confocal microscopy (Figure S1) but were not associated with defective Paneth cell development or numbers of Paneth cells (Figure S1B). Murine Ang4 is a member of the pancreatic ribonuclease superfamily that possesses poor angiogenic but strong microbicidal activity [44,47]. Using immunoblotting no Ang4 protein was detected in extracts of TCRδ^-/-^ intestinal mucosa (Figure 1C). Ang4 expression was further up‑regulated, although not significantly (p<0.06), in the small intestine of WT mice in response to oral infection by *Salmonella*. This increase did not occur in mice lacking γδ iIELs (Figure 1B). We have no evidence of altered Ang4 production in TCRδ^-/-^ mice other than in the small intestine and in Paneth cells (unpublished observations).

iIEL reconstitution experiments showed that Vγ7^+^ but not Vγ7^-^ iIELs from WT donors were sufficient to restore mRNA levels of Ang4 to WT levels in TCRδ^-/-^ recipients (Figure 1D and E). Of note, iIEL reconstitution did not affect the mRNA levels of other reference AMPs (Figure 1E) consistent with the RNA profiling data showing that the levels of other AMPs are unaffected by the absence of γδ T cells in TCRδ^-/-^ mice (Figures 1A and S1C). 

### γδ iIELs are involved in the initial host response against *Salmonella*

**Figure 2:**
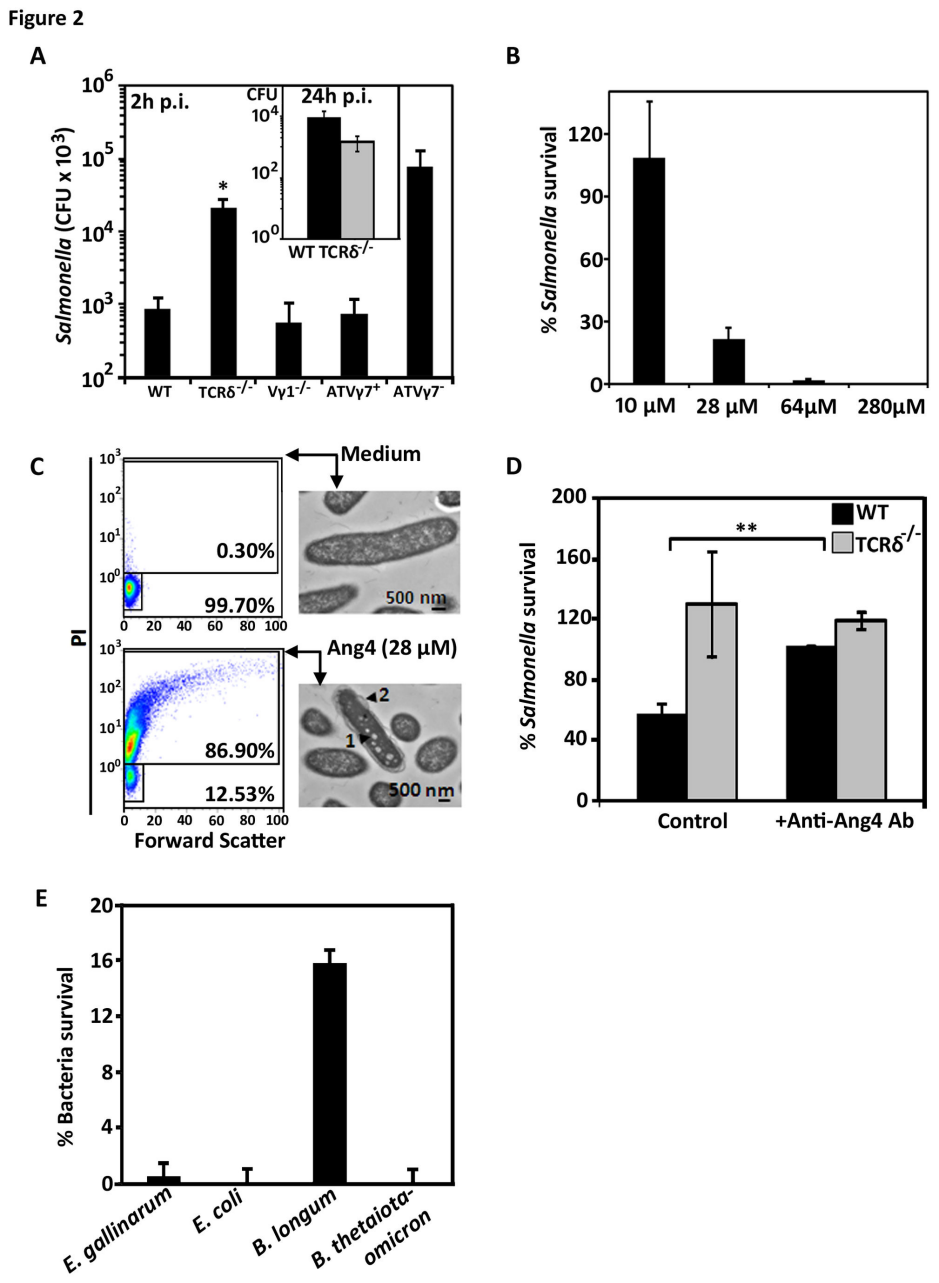
Vγ7^+^ iIELs are involved in resisting *Salmonella* invasion. (A) SL1344-Tn*lux* *Salmonella* population levels in the intestinal (ileal) mucosa of WT, TCRδ^-/-^ and TCRVγ1^-/-^ mice (n=10-12) and in TCRδ^-/-^ mice reconstituted with either Vγ7^+^ iIELs (ATVγ7^+^) or Vγ7^-^ iIELs (ATVγ7^-^) (n=5-6) 2h after oral challenge, (*p<0.01 comparing TCRδ^-/-^ with WT group). *Salmonella* CFU were quantified as described in the Materials and Methods. Inset: CFU of *Salmonella* SL1344wt strain in ileal mucosa 24h post-oral challenge. (B) Survival of 1x10^5^ *S*. Typhimurium SL1344 after 1h exposure to increasing amounts of recombinant Ang4, expressed as a percentage of population treated with PBS only. Data shown (mean±SEM) are representative of three independent experiments, each performed in triplicates. (C) Viability and cell membrane alteration of Ang4-treated *Salmonella* as assessed by PI staining and flow cytometry, and by transmission electron microscopy. The proportion of viable and dead bacteria after incubation with Ang4 or PBS is indicated by the % values shown in the quadrants. The TEM images are representative of 200‑300 *Salmonella* cells observed. The black arrowheads indicate regions of vesicle‑like structures (1) and blebbing of the outer membrane (2). (D) Survival of 1x10^5^ CFU *S*. Typhimurium SL1344 exposed to freshly collected TCRδ^-/-^ and WT crypt exudates in 10mM iPIPES (PIPES containing 137mM NaCl) in presence or absence of anti-Ang4 neutralising antibody (M20; Santa Cruz) (mean±SEM; **p≤0.005). Survival to Ang4 exposure was measured relative to that in non-treated exudates. (E) Survival of 1x10^5^ *Enterococcus* *gallinarum*, *Escherichia* *coli*, *Bifidobacterium* *longum* and *Bacteroides* *thetaiotaomicron* commensal bacteria after 1h exposure to 28μM of recombinant Ang4, expressed as a percentage of population treated with PBS only. Data shown are the mean±SEM of three independent experiments.

As γδ (Vγ7^+^) iIELs are necessary for the production of Ang4 (Figure 1A, B and E), we determined whether these cells could impact upon pathogen invasion of the intestinal mucosa through the control of Ang4 production, as shown for the intestinal parasite *Trichuris muris* [48]. During the first 2h of infection, the intestinal tissue (ileum) of mice lacking γδ iIELs was colonised by approximately 30 times more *Salmonella* than WT mice (Figure 2A). However, in TCRδ^-/-^ mice reconstituted with Vγ7^+^ iIELs the intracellular population levels of *Salmonella* were brought back to levels similar to those observed in WT mice (Figure 2A). By contrast, in TCRδ^-/-^ mice reconstituted with Vγ7^-^ iIELs the levels of *Salmonella* were comparable to those seen in non-manipulated TCRδ^-/-^ mice (Figure 2A). Of note, at later time points post infection, differences in the levels of *Salmonella* associated with small intestinal tissue (Figure 2A Inset) between WT and TCRδ^-/-^ mice were no longer apparent, consistent with recent findings [20] and suggesting that γδ iIELs act in the initiating of the host response.

Ang4 possesses bactericidal activity against the entero-pathogens *L. monocytogenes* and *E. faecalis* [44]. Using the same protocol as Hooper and colleagues [44] we determined whether Ang4 could kill *Salmonella* using recombinant Ang4. Recombinant Ang4 was active against *Salmonella* in a concentration‑dependent manner (Figure 2B); using amounts (280μM) comparable to those estimated to be present in the crypt lumen (Table 1) (~300μM), 100% of the *Salmonella* inoculum was killed (Figure 2B). Ang4 strongly affected bacterial membrane integrity with nearly 90% of the bacterial population that had been exposed to only 28μM Ang4 staining positive for propidium iodide (Figure 2C). Transmission electron micrographs confirmed abnormal cell structure of Ang4‑treated bacteria with affected cells exhibiting pore‑like structures in the cell membrane (Figure 2C). Native Ang4 in intestinal crypts was also active. Freshly obtained WT crypt exudates killed up to 70% of *Salmonella*, a significant proportion of which (~45%) could be attributed to Ang4 killing activity as seen by the reduction of killing in the presence of a neutralising anti-Ang4 monoclonal antibody (Figure 2D). This indicates that Ang4 produced *in vivo* remains active under conditions that reflect luminal electrolyte composition. As expected, no bactericidal activity was observed in TCRδ^-/-^ crypt exudates. As we speculated that Ang4 also contributes to the homeostasis of the intestinal microbial ecology, we tested whether Ang4 was also active against commensal bacteria using a selection of Gram‑positive and Gram‑negative strains from the sub‑ and predominant intestinal microbiota. Gram‑negative *Escherichia coli* and *Bacteroides thetaiotaomiron* were highly susceptible to Ang4 with less than 0.005% of the population surviving one-hour exposure to 28μM Ang4 (Figure 2E). Gram‑positive strains were also susceptible to Ang4 killing activity but to a lesser extent than the Gram‑negative strains tested, with approximately 0.5 and 16% of the population of *Enterococcus gallinarum* and *Bifidobacterium longum* surviving, respectively (Figure 2E). Overall, the high susceptibility to Ang4 killing activity of the commensals strains tested is consistent with Ang4 contributing to intestinal microbial homeostasis. Our findings also extend the known spectrum of Ang4 bactericidal activity to include *Salmonella* and intestinal commensal strains. Next we investigated how γδ iIELs modulate Ang4 expression.

**Table 1:** Estimated Ang4 content of mouse small intestinal crypts.

**Secretory stimulus** ^a^	**Ang4 content**	**Crypt Ang4**
	**ng/crypt ± SD**	**Concentration mM**
Non-stimulated	0.025 ± 0.014	0.30
IL-22 (100ng/ml)	0.175 ± 0.073	3.32
*Salmonella* (10^3^ CFU)	0.075 ± 0.016	1.51
Carbamyl Choline (10mM)	0.307 ± 0.064	4.38

^a^ Secretions collected after stimulation at 37°C for 30min

### γδ T cells are the major source of secreted factors necessary for increasing Ang4 production

**Figure 3:**
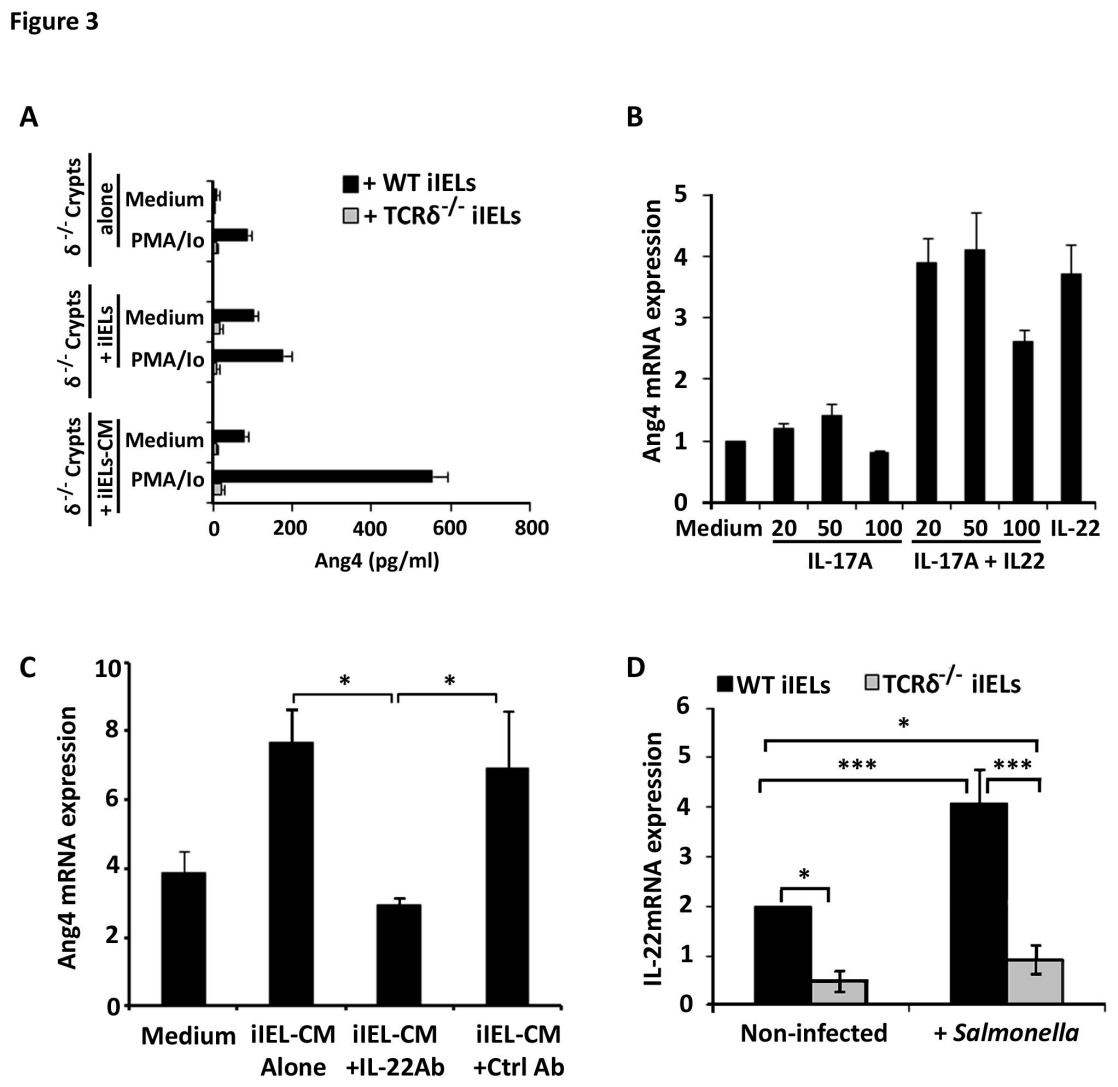
Paneth cells produce Ang4 in response to IL‑22 of which γδ iIELs are a source. (A) ELISA-determined Ang4 protein levels produced by small intestinal crypts (2x10^3^) from TCRδ^-/-^ mice after culture at 37°C for 4h in media alone (Medium) or in media containing PMA/Io. Additional crypt samples were cultured with 10^3^ WT iIELs (+iIELs) with and without prior *in* *vitro* stimulation by PMA/Io or, with conditioned medium (iIEL-CM) obtained from 10^4^ *in* *vitro* stimulated WT (black bars) and TCRδ^-/-^ (grey bars) iIELs. Data (mean±SEM) were collated from three experiments. (B) Ang4 mRNA levels (qPCR) detected in isolated intestinal TCRδ^-/-^ crypts/Paneth cells incubated at 37°C for 4h with recombinant murine IL‑17A alone or in combination with recombinant IL‑22 (100ng/ml). Control cultures contained medium or 100ng/ml IL-22 alone. Data (mean±SEM) are expressed relatively to mRNA levels obtained when crypts were exposed to medium alone and were collated from two experiments (see also Figure S2). (C) Anti-IL‑22 antibodies abrogate Ang4 expression by iIEL-CM. Small intestinal crypts from TCRδ^-/-^ mice were cultured at 37°C for 4h with iIEL-CM in the presence or absence of neutralising anti-IL‑22 or control antibodies (Ctrl Ab). Crypt Ang4 mRNA levels were measured by qPCR and expressed relative to β‑actin mRNA, with values (mean±SEM) representative of three experiments. (D) IL‑22 expression is reduced in TCRδ^‑/-^ iIELs. IL‑22 mRNA was isolated from iIELs of WT and TCRδ^-/-^ mice prior to (Non-infected) and 2h post‑challenge with *Salmonella* by qPCR. The data (mean±SEM) are expressed relative to β‑actin mRNA and were collated from four experiments (*p≤0.05; ***p≤0.001).

An iIEL-intestinal crypt/Paneth cell co-culture system was developed to determine the role γδ iIELs play in inducing Ang4 production by Paneth cells and whether it requires cognate or non-cognate interactions. Intestinal crypts from TCRδ^‑/-^ mice were used as they produce no or undetectable amounts of Ang4 (Figure 1B and C). The addition of WT iIELs to TCRδ^‑/-^ intestinal crypts resulted in small increases in Ang4 protein (Figure 3A) and mRNA (Figure S3A) expression with iIELs expressing no Ang4 expression themselves (data not shown and [44]). By contrast, supernatants of *in vitro* activated WT iIELs (IEL-CM) induced up to 10 and 50 times more Ang4 mRNA and protein production compared to crypts cultured in medium alone, respectively (Figure 3A, S3A). No significant stimulatory activity of Ang4 production was observed in control cultures containing the chemical stimuli used to activate iIELs *in vitro* or using supernatants from activated TCRδ^-/-^ iIELs. Thus, γδ iIELs are a source of factors that up‑regulate Ang4 expression, requiring no direct interaction with crypt/Paneth cells for Ang4 production.

### IL‑22 is necessary and sufficient for up‑regulating Ang4 expression

IL‑22 and IL‑17 are required for the production of AMPs in the skin and the inflamed intestine [49-54] with activated T lymphocytes being a source of both [55,56]. We therefore tested the possibility that IL-17 and IL-22 might regulate Ang4 production by Paneth cells. Recombinant IL‑22 up‑regulated Ang4 expression in isolated intestinal crypts from TCRδ^‑/-^ mice whereas IL‑17 alone or in combination with IL‑22 had no significant effect (Figure 3B and S3B). By contrast, IL‑22 did not affect the production of any of the other Paneth cell AMPs tested here, including lysozyme, (Figure S1C and data not shown). The addition of neutralising anti‑IL‑22 antibodies to supernatants from activated WT iIELs inhibited their ability to up‑regulate Ang4 mRNA expression in Paneth cell‑containing crypts (Figure 3C), suggesting that IL‑22 is both necessary and sufficient to increase Ang4 expression in Paneth cells. Furthermore, using an *ang4* promote*r::luc* reporter system, IL‑22 was shown to act at the transcriptional level on Ang4 expression in m-ICc12 epithelial cells (Figure S4). iIELs isolated from TCRδ^-/-^ animals expressed significantly lower levels of IL‑22 mRNA compared to those of both naïve and infected WT mice (Figure 3D). IL‑22 mRNA was more abundant in γδ^+^ than in αβ^+^ iIELs (Figure S5A) and the majority (75%) of IL-22 secreting iIELs from *Salmonella* infected WT mice were TCRγδ^+^ (Figure S5B) of which 75% were Vγ7^+^ (Figure S5C). 

### iIELs produce IL‑22 in response to IL‑23 produced by stressed intestinal epithelial cells

**Figure 4:**
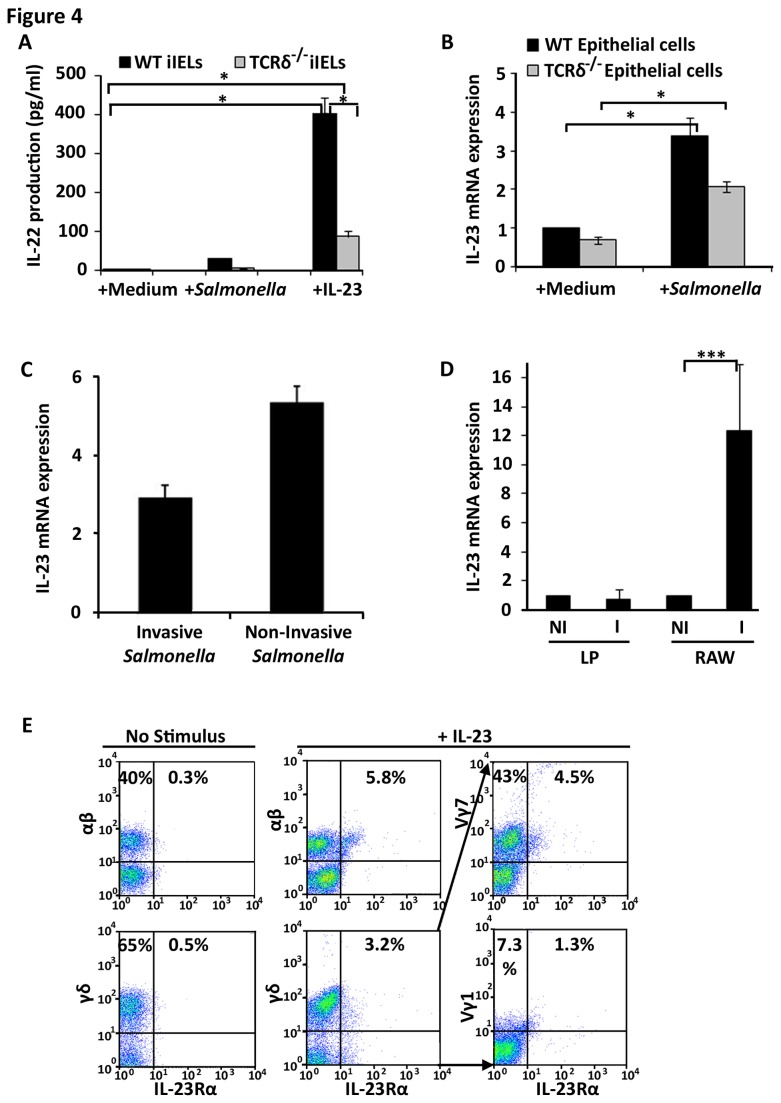
iIELs produce IL-22 in response to IL-23, a cytokine produced by intestinal epithelial cells. (A) iIELs from WT and TCRδ^‑/‑^ mice were cultured at 37°C for 6h with medium alone or medium containing 1x10^5^ CFU *S*. Typhimurium or recombinant IL‑23 (100ng/ml) after which supernatants were analysed by IL‑22 ELISA (*p≤0.05). Data (mean±SEM) were collated from three experiments. (B) Intestinal epithelial cells (10^6^) from WT and TCRδ^‑/-^ mice were exposed for 2h to medium alone or medium containing *Salmonella* at a ratio of 10 bacteria per epithelial cell. IL‑23 mRNA expression was then assessed by qPCR. The data (mean±SEM) were expressed relative to mRNA levels found in WT epithelial cells exposed to medium alone and were collated from three experiments (*p≤0.05). (C) m-ICcl2 intestinal epithelial cells were cultivated on 0.4µm pore size filters and incubated at 37°C for 4h with medium alone, or medium containing invasive or non‑invasive *Salmonella* after which cells were harvested, RNA extracted and IL‑23 expression analysed by qPCR. Data are expressed relative to mRNA levels obtained on m‑ICc12 cells exposed to medium alone. (D) RNA was purified from lamina propria (LP) cells isolated from orally infected (2h p.i.) and non‑infected WT mice. As a positive control for IL-23 production, cultivated RAW264.7 macrophages (RAW) were analysed pre- (NI) and post-infection (I) (6h p.i.) with *Salmonella*. Data are shown as mean±STD relative to mRNA levels in non‑infected samples, and are representative of 5 and 4 experiments, respectively. (E) iIELs from WT mice were cultured at 37°C for 2h with medium alone (No Stimulus) or with medium containing IL‑23. The distribution of IL-23Rα expression among Vγ1 and Vγ7 iIELs was determined by flow cytometry from 4-colour/antibody staining protocols: combining CD3, TCR-γδ, IL-23R and either TCR-Vγ7 or TCR-Vγ1 antibodies with a gating strategy of selecting CD3^+^, TCR-γδ^+^, IL-23R^+^ events (middle panels) and then analysing them for Vγ7^+^, IL-23Rα^+^ and Vγ7^-^, IL-23Rα^+^ events (far right hand panels). The profiles shown are representative of three experiments with the percentage values representing the frequency of positive cells in the designated quadrants.

The finding that IL‑22 production by γδ iIELs is increased upon *Salmonella* infection suggests that γδ iIELs either directly interact with *Salmonella* and secrete IL‑22, as shown for lung γδ IELs exposed to *Bacillus subtilis* [57], or that local pathogen-exposed epithelial cells produce signalling molecules able to trigger IL‑22 production by iIELs. Several studies have previously demonstrated that IL‑23 regulates mucosal inflammation induced by pathogen infection such as *Salmonella* [58,59]. IL‑23 has also been shown to induce IL-22 production by Th17 cells and peripheral γδ T cells [60]. The possibility that IL-22 production by iIELs is regulated by IL‑23 was determined by exposing highly purified WT and TCRδ^-/-^ iIELs to *Salmonella* or recombinant IL‑23. Exposure to *Salmonella* increased the levels of IL‑22 produced by WT iIELs with IL‑23 having an even greater effect (Figure 4A), leading to a ~400‑fold increase in the amount of IL‑22 protein detected compared to that produced by iIELs exposed to medium alone. By contrast, IL‑23 induced much lower levels of IL‑22 among TCRδ^-/-^ iIELs, consistent with γδ^+^ iIELs being the major source of IL‑22 among iIELs.

IL‑23 is expressed by macrophages and other professional phagocytes upon pathogenic or commensal challenges [61-64] although non-phagocytic cells are now being identified as additional sources of IL‑23 [65]. We therefore tested the possibility that intestinal epithelial cells are an immediate source of IL-23 following challenge with *Salmonella*. Upon a 4h exposure of primary small intestinal epithelial cells (>99% CD45^-^; Figure S6) or m‑ICc12 cells to *Salmonella* the levels of IL‑23 mRNA significantly increased in both cell types (Figure 4B and C). *Salmonella* induced slightly lower levels of IL-23 mRNA in epithelial cells from TCRδ^-/-^ mice compared to WT cells which we cannot explain but may reflect the absence of γδ iIELs *in vivo* and perhaps their requirement for optimal epithelial cell responses. Of note, up‑regulation of IL‑23 expression in m‑ICc12 epithelial cells was also observed in response to non‑invasive *Salmonella* (Figure 4C), suggesting that invasion was not required to trigger the IL‑23‑mediated epithelial response.

The contribution of other, non-epithelial, intestinal mucosal cells to IL-23 production during the initial response to oral *Salmonella* infection was investigated by examining IL-23 mRNA expression by mucosal immune cells directly *ex‑vivo* during the first 2h of infection. Under these experimental conditions it was not possible to detect IL-23 expression by these cells (Figure 4D). However, these lamina propria cells are capable of expressing IL‑23 when directly challenged in isolation after exposure to *Salmonella in vitro* for 4h, as done for primary and m‑ICc12 epithelial cells (Figure S7). In response to IL-23 iIELs rapidly expressed IL-23Rα; approximately 10% of iIELs (~6% TCR-αβ^+^ and ~3% TCR-γδ^+^) expressed IL‑23Rα within 2h of *in vitro* stimulation which represents a 6- to almost 20-fold increase in IL‑23Rα^+^ iIELs when compared with IELs exposed to medium alone (Figure 4E). The majority of IL‑23Rα^+^ cells were γδ iIELs of which >43% were Vγ7^+^, consistent with this subset being instrumental in the IL‑23‑dependent signalling cascade leading to Ang4 expression (Figure 4E). Naive iIELs were virtually devoid (<1% positive) of IL‑23Rα expression (Figure 4E). 

### Invasion of intestinal epithelial cells by Salmonella is not essential for up‑regulation of IL‑23 by intestinal epithelial cells and Ang4 production by Paneth cells

**Figure 5:**
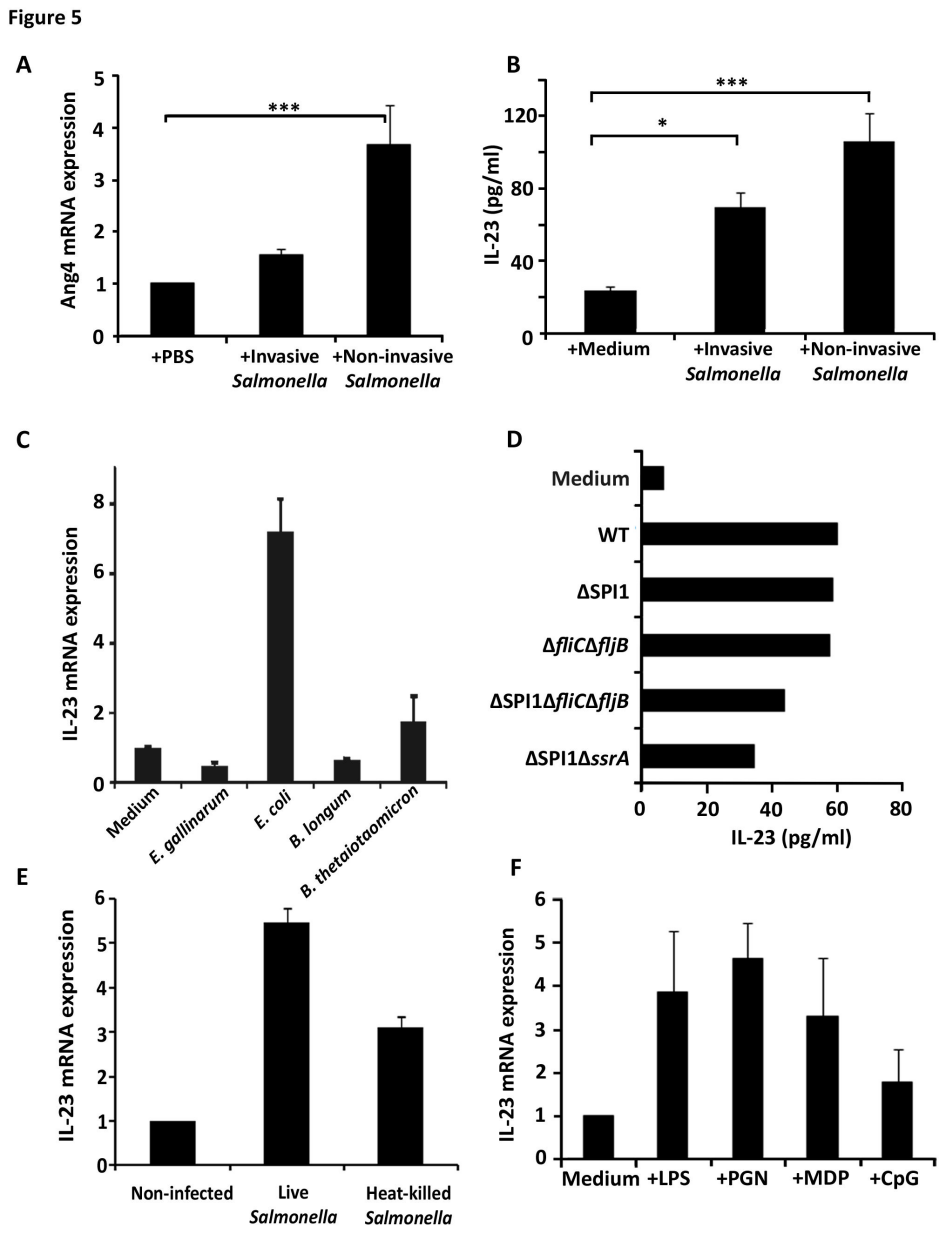
Requirements for microbe-induced Ang4 and IL‑23 production by intestinal epithelial cells. (A) Invasive or non-invasive *Salmonella* strains (4x10^7^ CFU in PBS) were injected into exteriorised intestinal ligated loops of WT mice. Four hours later mucosal RNA was isolated and Ang4 mRNA expression analysed by qPCR. The data (mean±SEM) are expressed relative to mRNA levels obtained from loops exposed to PBS alone (n=3; ***p<0.001). (B) IL‑23 protein levels assessed by ELISA (mean±SEM) from intestinal epithelial cells of WT mice exposed for 12h to medium alone, (+Medium) or media containing invasive or non‑invasive *Salmonella* strains at a ratio of 10 bacteria per epithelial cell (n=3; ***p<0.001, *p<0.05). (C) m-ICc12 intestinal epithelial cells were cultured for 4h with medium alone (control) or containing live cells of various intestinal commensal bacteria at a ratio of 10 bacteria per epithelial cell. IL‑23 mRNA expression was assessed by qPCR. Data (mean±SEM) were expressed relatively to mRNA levels in control samples (n=4). (D) IL‑23 protein production measured by ELISA in intestinal epithelial cells from WT mice cultured at 37°C for 12h with medium alone or containing either, live WT (SL1344) or various *Salmonella* mutant strains that are non-invasive (SL1344ΔSPI1), invasive but non-flagellated (JH3220=SL1344Δ*fliC*Δ*fljB*), non-invasive and non-flagellated (JH3515=SL1344∆SPI1Δ*fliC*Δ*fljB*) or non-invasive, flagellated but unable to transcytose flagellin (JH3574=SL1344∆SPI1Δ*ssrA*) (n=3; mean±SEM). (E) IL‑23 mRNA expression assessed by qPCR in m-ICc12 epithelial cells cultured at 37°C for 4h with medium alone or containing live or heat‑killed WT *Salmonella* SL1344 cells at a ratio of 10 bacteria per epithelial cell. Data (n=4; mean±SEM) are expressed relative to mRNA levels obtained in non‑infected cells. (F) IL‑23 mRNA levels analysed by qPCR in intestinal epithelial cells from WT mice cultured at 37°C for 2h with medium alone or containing lipopolysaccharide (LPS), peptidoglycan (PGN), muramyl dipeptide (MDP) or methylated DNA (CpG). Data (n=3; mean±SEM) are expressed relative to mRNA levels obtained in cells exposed to medium alone.

The relationship between IL‑23 expression by intestinal epithelial cells after oral challenge by *Salmonella* and Ang4 expression by Paneth cells was investigated *in vivo*. *Salmonella* was injected into ligated loops of small intestine in WT mice, resulting in increased levels of Ang4 mRNA irrespective of whether *Salmonella* was invasive or not (Figure 5A). Similarly, IL‑23 secretion was increased in cultured intestinal epithelial cells after challenge with both invasive and non-invasive *Salmonella* strains (Figure 5B), with up‑regulation of Ang4 *in vivo* and of IL‑23 *in vitro* surprisingly being 1.5 to 2‑fold higher when non‑invasive *Salmonella* was used. Although difficult to explain, these results in combination with the bactericidal activity spectrum observed (Figure 2) raised the possibility that the IL‑23/IL‑22/Ang4 pathway is also active in response to non‑invasive bacteria such as components of the intestinal microbiota. Gram-negative *E. coli* and to a lower extent *B. thetaiotaomicron* triggered IL‑23 mRNA expression in intestinal epithelial cells (Figure 5C) whereas two Gram-positive bacteria tested, *E. gallinarum* and *B. longum*, failed to invoke an IL‑23 response from epithelial cells, although they are susceptible to Ang4 activity (Figure 2E). Overall these results suggest that the pathway described here may contribute to the host-microbe homeostasis of the GI tract. 

To understand in more details the pathway leading from bacterial challenge to IL‑23 production, intestinal epithelial cells from WT mice were exposed to a virulent strain of *Salmonella* or *Salmonella* strains lacking various virulence factors including flagellin, invasion‑associated pathogenicity island (*Salmonella* Pathogenicity Island 1, SPI1), or SsrA, which is a key regulator of the SPI2 (essential for intracellular survival, replication and flagellin translocation across the epithelial layer). Irrespective of whether or not bacteria could invade, synthesise or transcytose flagellin, all mutant strains induced IL‑23 secretion by primary epithelial cells (Figure 5D), suggesting that the three key virulence gene clusters were not required for IL‑23 up‑regulation. In general agreement with this, we observed a trend of increased numbers of bacteria (~5-fold) in the small intestinal tissue of TCRδ^‑/-^ mice compared to WT mice after 2h infection with two mutant, non-flagellated and non‑invasive, strains of *Salmonella* (SL1344Δ*fliC*Δ*fljB* = 7.47x10^3^±1.53x10^3^ CFU/g in WT mice compared with 3.14x10^4^±9.18x10^3^ CFU/g in TCRδ^‑/-^ mice; SL1344ΔSPI1 = 5.09x10^3^±3.93x10^3^ CFU/g in WT mice compared with 2.32x10^4^± 1.80x10^4^ CFU/g in TCRδ^‑/-^ mice). Heat-killed *Salmonella* cells also induced IL‑23 mRNA expression by m‑ICc12 cells (Figure 5E), suggesting that bacterial cell components, likely to be surface‑exposed were sufficient to elicit IL‑23 expression. To test this hypothesis purified microbe‑associated molecular patterns (MAMPs) including LPS, Peptidoglycan and muramyl dipeptide (MDP) were applied for up to 16h to cultured intestinal epithelial cells. All bacterial components (MDP to a lesser extent than others) triggered up‑regulation of IL-23 mRNA expression in intestinal epithelial cells (Figure 5F). By contrast, the TLR9 ligand CpG did not affect IL‑23 production and the ability of non-flagellated *Salmonella* to invoke IL-23 production by epithelial cells excluded TLR5 involvement in this response. These findings suggest that recognition of *Salmonella* by epithelial cells is mediated by multiple MAMPs recognised by epithelial cell surface or cytoplasmic pattern recognition receptors (PRRs).

## Discussion

**Figure 6:**
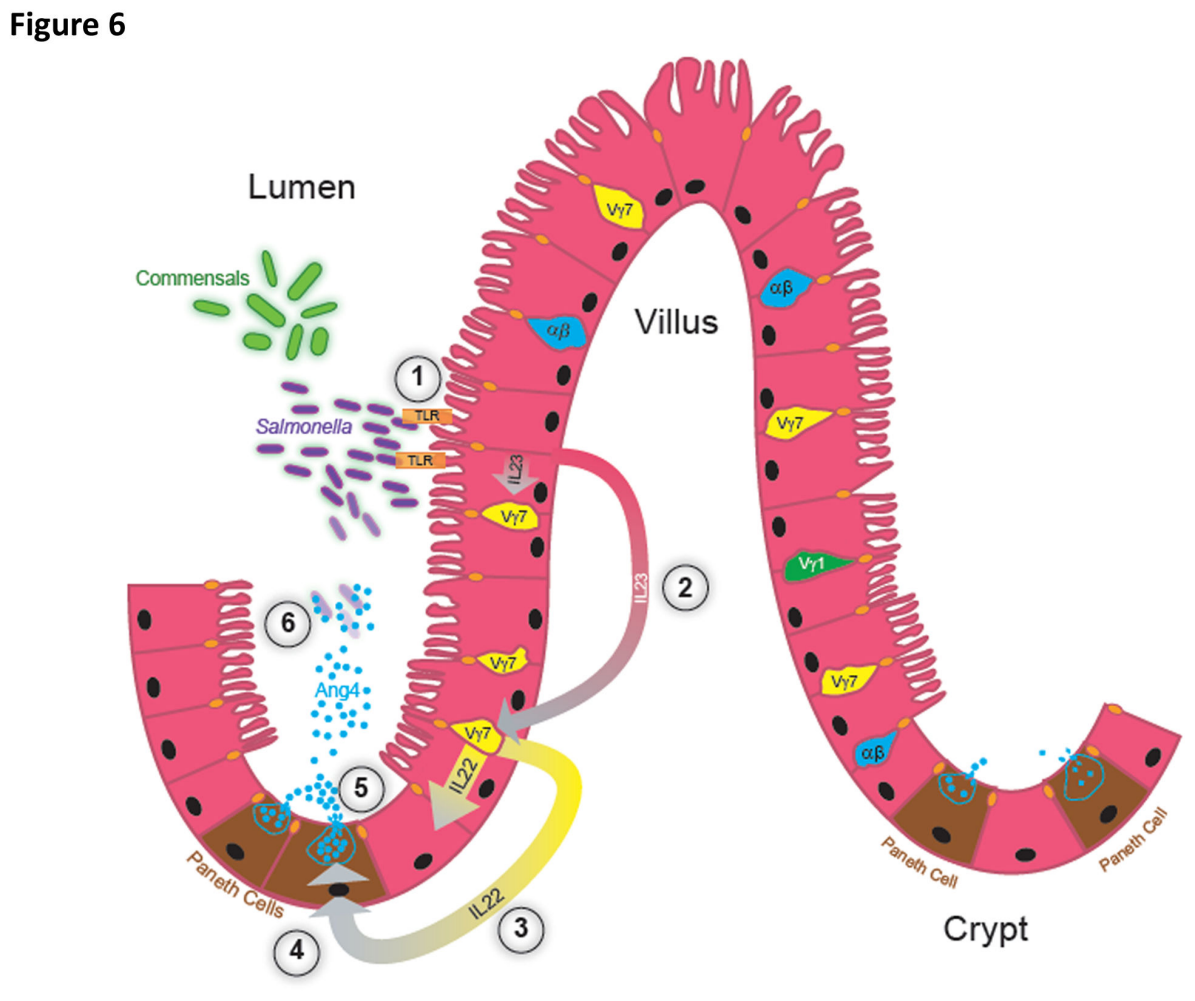
Hypothetical model of the production of Ang4 in response to microbial challenge. (1) Upon exposure to *Salmonella* or recognition of commensal bacteria or MAMPs, intestinal epithelial cells secrete IL‑23, in a TLR‑dependent manner in the case of *Salmonella*. (2) Via extracellular or transcellular routes, epithelial cells secrete IL‑23 (pink to grey gradient arrows) that binds to its cognate receptor IL‑23R expressed by γδ iIELs. (3) IL-23R^+^ iIELs enriched in Vγ7^+^ cells respond to IL-23 by secreting IL‑22 (yellow to grey gradient arrows). Via extracellular or transcellular routes IL‑22 acts on IL-22R-bearing Paneth cells up-regulating Ang4 transcription (4) and/or secretion (5) of pre-formed protein stored in intracellular granules. Ang4 is secreted into the lumen at levels sufficient (5) to effectively kill *Salmonella* located in the vicinity of the intestinal tissue (6), helping to protect it from proliferation of and invasion by the pathogen.

The intimate association of TCRγδ^+^ iIELs with the epithelium of the small intestine is indicative of their involvement in maintaining intestinal barrier function. Exactly how γδ iIELs fulfil this role, the nature of their interactions with the epithelium, and the pathways leading to their activation and deployment of effector function is unclear. Here, we describe a new putative host response pathway involving the interaction of γδ (Vγ7^+^) iIELs with epithelial cells leading to Ang4 production by Paneth cells, using *Salmonella* as a model pathogen to trigger this response *in vivo* (Figure 6). Of note, commensal members of the intestinal microbiota also appear to have the capacity to trigger this pathway. While we can provide no evidence that this pathway is directly involved in determining the outcome of oral *Salmonella* infection and the development of salmonellosis, it has the potential to act in synergy with or in addition to other mechanisms leading to AMP production, to reduce the level of host cell infection by pathogens as well as proliferation of commensals in close vicinity to the epithelium and maintaining the integrity of the intestinal epithelial barrier. 

Commensal intestinal bacteria influence both Ang4 production and iIELs. Ang4 expression increases dramatically in ex‑germfree mice in response to colonisation by gut bacteria [44] and iIEL populations require exposure to environmental antigens and commensal bacteria to become functionally mature [66,67]. Our finding that Ang4 production is initiated by epithelial non‑clonotypic PRRs and that both commensals and pathogens can elicit the pathway leading to Ang4 production [44] demonstrate a lack of distinction in the origin of bacteria that triggers Ang4 expression. Our data does however suggest that Gram-negative bacteria are more effective at triggering IL‑23 expression, and are noticeably more susceptible to Ang4 than Gram‑positive bacteria. Depending on the diffusion gradient of secreted Ang4 in the crypt/villous lumen it may impact on microbes within the mucus layer covering the epithelium or on microbes able to penetrate the mucus layer, which has a thinner viscosity at the bottom of crypts [68] thereby preventing microbial access to the underlying epithelium and resulting in limited activation of inflammatory responses. Our data suggest that the ability of bacteria or bacterial cell components to make contact with surface epithelial cells and elicit PRR-mediated IL‑23 production could be the driving force behind epithelial cell interactions with IL‑22‑producing Vγ7^+^ iIELs that subsequently leads to Ang4 production. This bacteria-driven response may serve the host by containing and restricting the growth of populations of commensal bacteria at the luminal surface and by containing the growth of invasive pathogens that encroach into intestinal crypts. It was technically not possible to reconstitute Ang4 activity in TCRδ^-/-^ mice by enteric delivery of native, highly labile, Ang4 (data not shown) to determine whether the lack of Ang4 in TCRδ^‑/-^ mice directly accounted for the higher *Salmonella* burden in intestinal tissue immediately after oral challenge. However, we have shown that Ang4 produced *in vivo* is bactericidal in intestinal crypt exudates. *Salmonella* is unlikely to be the only target of Ang4 bactericidal activity and it may target other enteric bacterial pathogens including *Listeria* [44] to which TCRδ^-/-^ mice display increased susceptibility [26]. Ang4 can therefore be considered part of the AMP armament that protects the host against invasion by a range of enteric microbial challenges.

How γδ T cell responses are triggered and the nature of the antigens they recognise are uncertain. This study shows that their activation involves signals delivered through cytokine-cytokine receptor axes, in particular IL‑23/IL‑23R signalling, extending previous reports of γδ T cell involvement in IL‑23‑orchestrated mucosal responses to bacteria [62] or to *Salmonella* [69]. Our findings clearly show that IL-23 alone is sufficient to elicit IL-22 production by cultured γδ iIELs, consistent with the lack of evidence of a requirement for cognate iIEL-Paneth cell interactions in Ang4 production. How accurately this reflects the requirements for γδ iIEL activation and IL-22 production *in vivo* requires further study. It is possible is that other mucosa-associated immune cells support γδ T cells in their IL-23-driven IL-22 production, as for example shown for populations of macrophages and Lymphoid inducer cells responding to *Citrobacter rodentium* infection [70,71]. The apparent lack of TCR involvement in γδ iIEL activation and IL‑22 production is similar to that described for splenic and lymph node γδ T cells that produce IL‑22 and IL‑17 in response to IL‑23 plus IL1 and TLR ligands [72,73], the cellular source of which includes activated dendritic cells [72]. During the immediate response to oral *Salmonella* challenge our results identify epithelial cells as the source of IL-23, although we do not exclude that at later times post-microbial challenge IL‑23 is expressed by innate immune cells present or recruited to the lamina propria, as has been described for other pathogens in different mucosal systems [74-76]. We cannot formally exclude the involvement of specific antigen receptors (TCR and PRRs) in iIEL responses to *Salmonella* infection *in vivo*. However, our findings exclude the TCR acting in isolation to trigger their response and suggest that TLR involvement in γδ iIELs activation and IL‑22 production is indirect and is consequent to upstream response of intestinal epithelial cells that rapidly produce IL‑23 in response to bacterial cells and/or their products. We cannot exclude that lamina propria immune cells are an additional and major source of IL‑23 later during infection. However, these cells did not up-regulate IL‑23 mRNA expression *in vivo* within the first 2h of infection whereas primary epithelial cells did in our *in vivo* and *in vitro* model systems. Interestingly, certain Gram-negative commensals such as *E. coli* elicited an IL-23 response from intestinal epithelial cells, similar in magnitude to that triggered by *Salmonella*. The IL‑23 signalling pathway may therefore not be microbe‑specific and unable to discriminate between pathogens and commensals. Instead, it may be part of a more generic homeostatic response aimed at controlling microbial proliferation in close vicinity of the intestinal mucosa. The apparent lack of TLR responsiveness and the absence of IL‑23R expression among γδ iIELs in naïve animals provide a means by which their activation can be regulated. Furthermore this can be coordinated through the response of epithelial cells, preventing them from responding directly to enteric microbes. The transient and low levels of IL‑23 produced by stressed/infected epithelial cells could act in a similar way to restrict and contain iIEL activation and the production of pro-inflammatory cytokines, which if sustained and uncoordinated could promote tissue damaging responses. Of note, patients with IL-12/IL-23 component deficiency are susceptible to recurrent *Salmonella* infection [77]. It would be of interest to determine if this is associated with abnormalities in iIEL and/or AMP function.

In the context of the initial response of γδ IELs to oral *Salmonella* exposure and intestinal epithelial stress, the findings presented here add on to previous studies showing that IL‑22 is of primary importance in host antimicrobial defence in ensuring the production of potent AMPs [54,78]. IL‑22 deficiency in patients with *acne inversa* associated with defective AMP production and bacterial overgrowth is consistent with this interpretation and the importance of IL‑22 in microbial defence at mucosal sites [79]. The level at which IL‑22 acts to regulate Ang4 secretion has not been fully established although the data presented here suggests that Ang4 production is controlled at the transcriptional level by iIELs and IL‑22. Although iIELs are a source of IL-17 that plays a central role in (inflammatory) mucosal immune responses it is not required for iIEL-driven Ang4 production. It may however contribute in other ways to reinforce barrier functions via, for example, its effects on mucin and other AMP production (Lipocalin 2) and epithelial tight junctions [52,80,81].

In summary, our findings identify a novel host response pathway triggered by the model enteric pathogen *Salmonella* and components of the microbiota that involves the coordinated interaction of γδ iIELs with microbe/pathogen exposed epithelial cells. This cross‑talk occurs through specific cytokine-cytokine receptor signalling, resulting in up‑regulation of Ang4 production by Paneth cells (Figure 6) that can contribute to maintaining intestinal microbial homeostasis and mucosal defence of the GI tract. 

## Supporting Information

Figure S1 **Paneth cell development and lysozyme production occurs normally in the absence of γδ T cells.**
(TIFF)

Figure S2 **Intestinal crypt isolation.**
(TIF)

Figure S3 **qPCR quantitation of Ang4 mRNA expression in small intestinal crypts.**
(TIFF)

Figure S4 **IL‑22 acts at the transcriptional level to regulate Ang4 expression.**
(TIFF)

Figure S5 **IL‑22 induces Ang4 mRNA and protein expression and is produced by TCRVγγ^+^ iIELs.**
(TIFF)

Figure S6 **Purity of intestinal epithelial cell preparations.**
(TIFF)

Figure S7 ***In vitro* stimulated lamina propria cells express IL‑23 mRNA.**
(TIFF)
