# Supplementary figures and images for: Intestinal Intraepithelial Lymphocyte-Enterocyte Crosstalk Regulates Production of Bactericidal Angiogenin 4 by Paneth Cells upon Microbial Challenge
Source: PLoS One. 2013 Dec 17;8(12):e84553. doi: 10.1371/journal.pone.0084553 (PMC3866140; doi:10.1371/journal.pone.0084553)

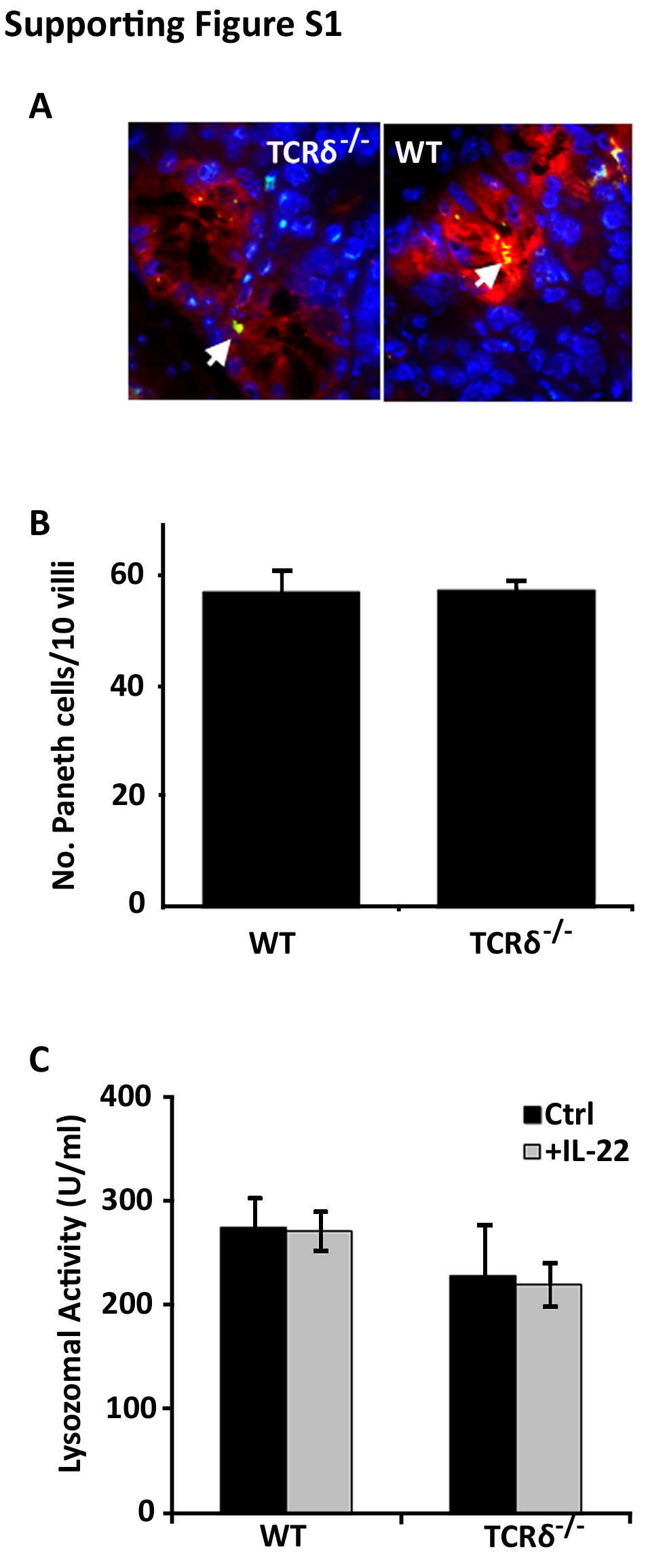

Supplement: Figure S1 — Paneth cell development and lysozyme production occurs normally in the absence of γδ T cells. (TIFF) [file pone.0084553.s001.tiff]

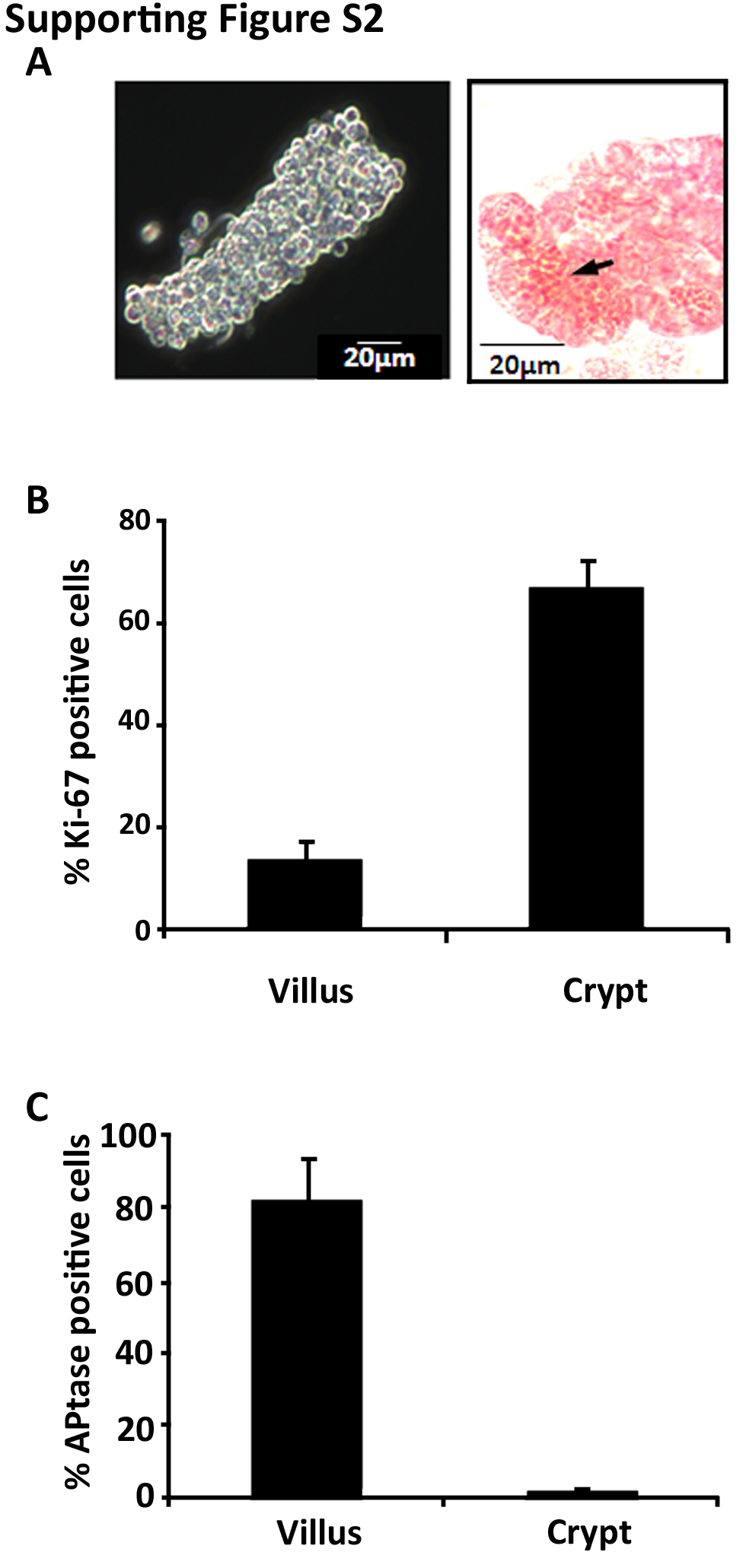

Supplement: Figure S2 — Intestinal crypt isolation. (TIF) [file pone.0084553.s002.tif]

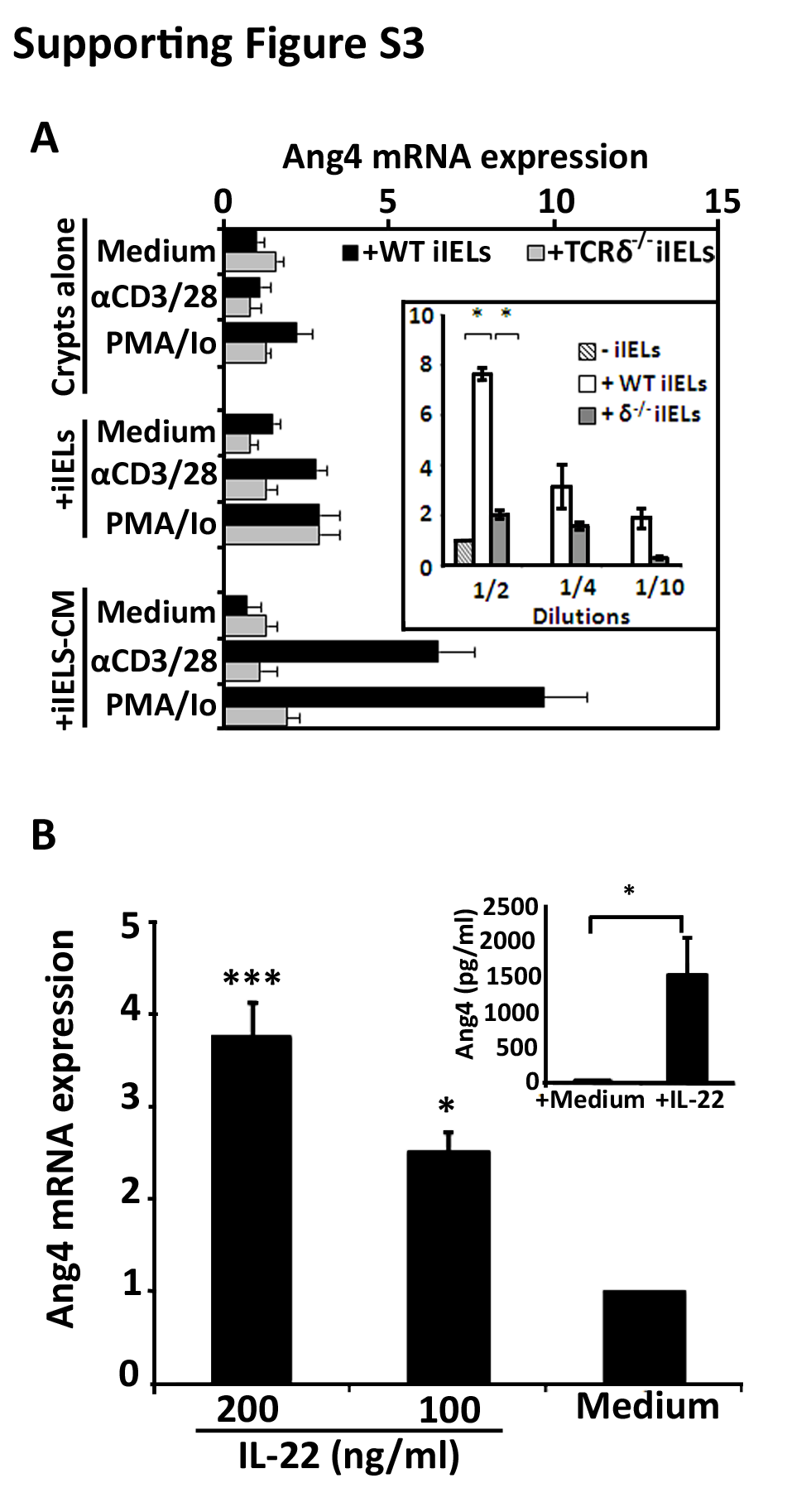

Supplement: Figure S3 — qPCR quantitation of Ang4 mRNA expression in small intestinal crypts. (TIFF) [file pone.0084553.s003.tiff]

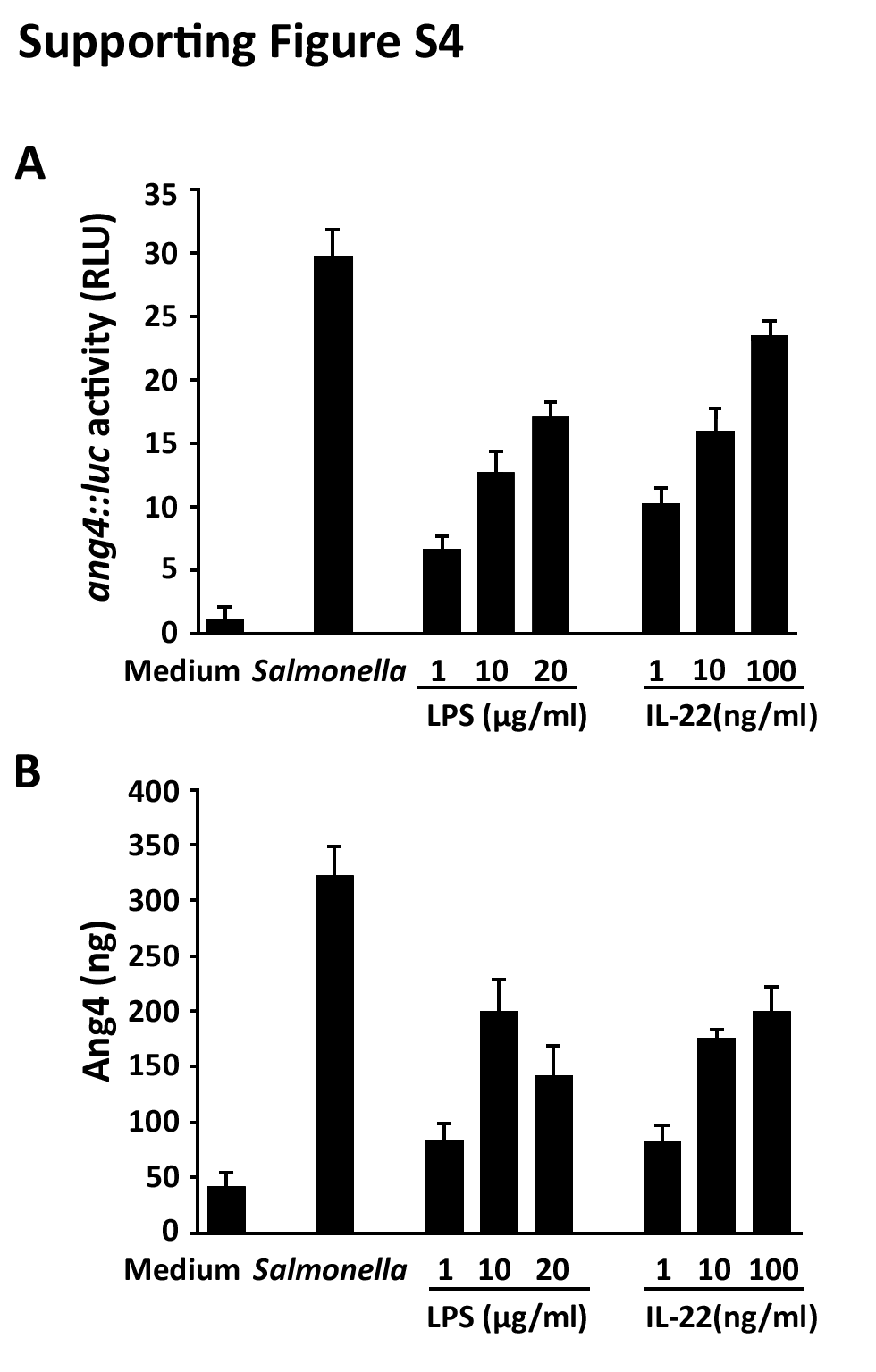

Supplement: Figure S4 — IL‑22 acts at the transcriptional level to regulate Ang4 expression. (TIFF) [file pone.0084553.s004.tiff]

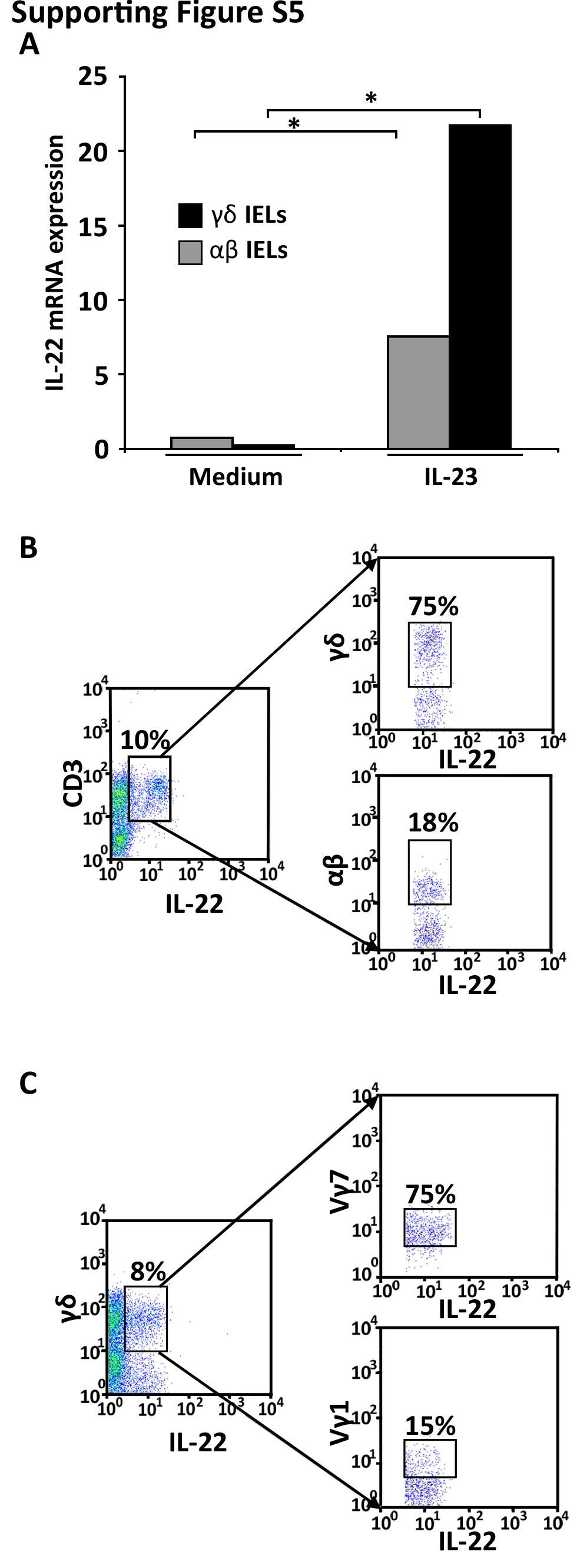

Supplement: Figure S5 — IL‑22 induces Ang4 mRNA and protein expression and is produced by TCRVγγ+ iIELs. (TIFF) [file pone.0084553.s005.tiff]

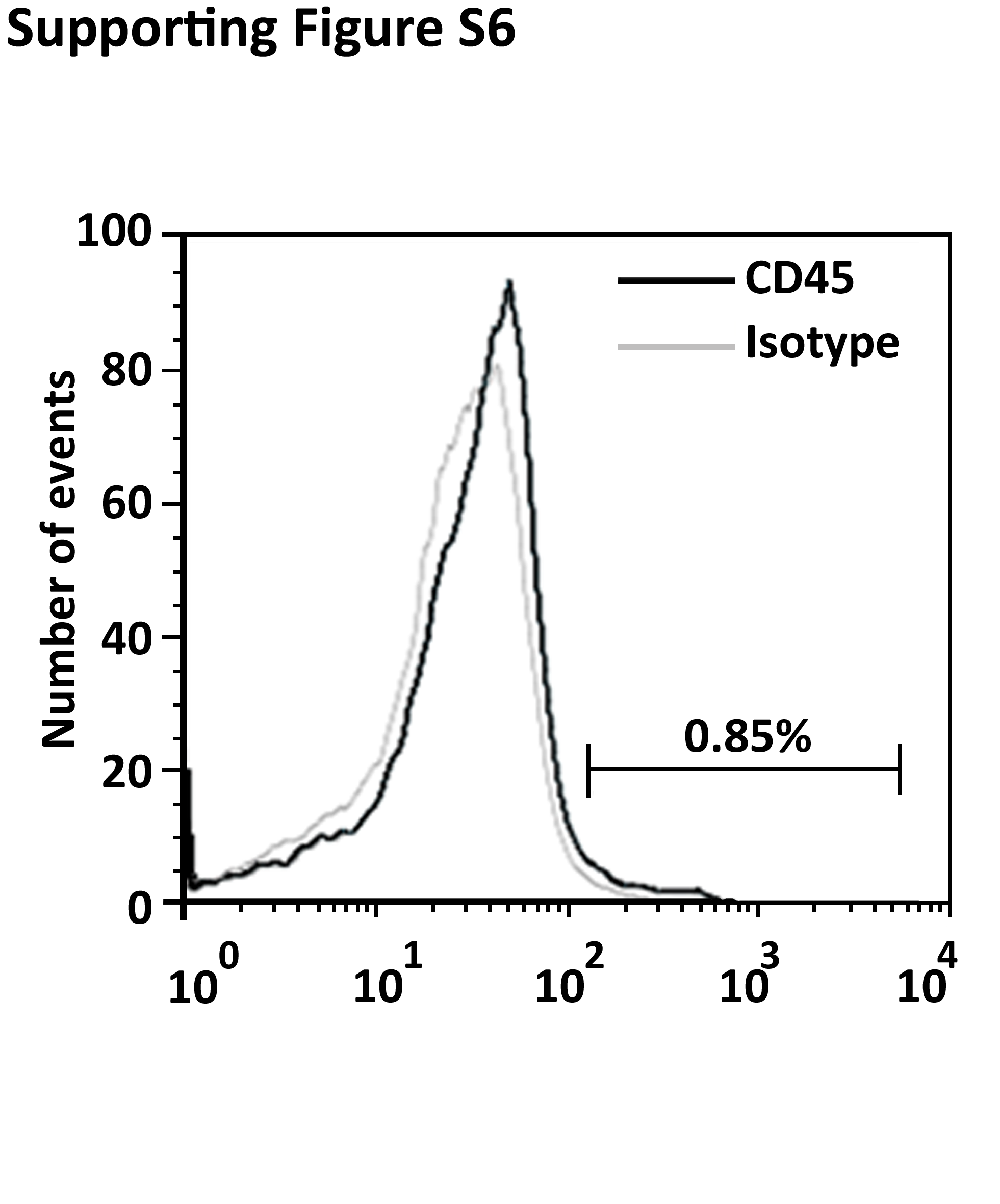

Supplement: Figure S6 — Purity of intestinal epithelial cell preparations. (TIFF) [file pone.0084553.s006.tiff]

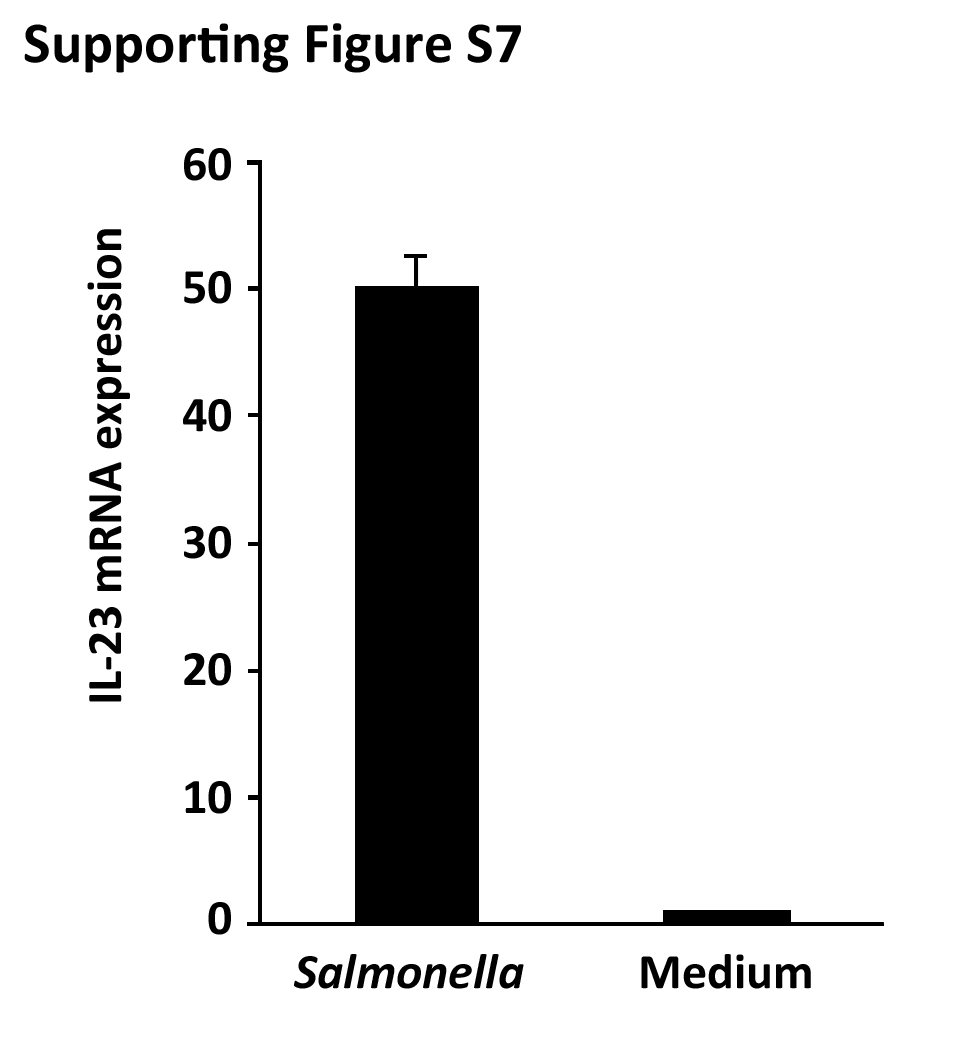

Supplement: Figure S7 — In vitro stimulated lamina propria cells express IL‑23 mRNA. (TIFF) [file pone.0084553.s007.tiff]
